# Neuroimaging Biomarkers in Neuropsychiatric Symptom Clusters

**DOI:** 10.1002/alz.087950

**Published:** 2025-01-09

**Authors:** Daniel Kapustin, Neda Rashidi‐Ranjbar, Wei Wang, Malcolm Binns, Paula M McLaughlin, Agessandro Abrahao, David A. Grimes, Anthony E Lang, Connie Marras, Mario Masellis, Joseph B Orange, Tarek K. Rajji, Angela C Roberts, Gustavo Saposnik, Richard H Swartz, David F. Tang‐Wai, Carmela Tartaglia, Angela Troyer, Lorne Zinman, Corinne E. Fischer, Sanjeev Kumar

**Affiliations:** ^1^ University of Toronto, Toronto, ON Canada; ^2^ Keenan Research Centre for Biomedical Science, the Li Ka Shing Knowledge Institute, St. Michael’s Hospital, Toronto, ON Canada; ^3^ Centre for Addiction and Mental Health, Toronto, ON Canada; ^4^ Baycrest Academy for Research and Education, Toronto, ON Canada; ^5^ Nova Scotia Health, Halifax, NS Canada; ^6^ Assistant professor, department of medicine (neurology), U of T, Toronto, ON Canada; ^7^ Neurologist Sunnybrook Health Sciences Centre, Toronto, ON Canada; ^8^ Neurology/Medicine, University of Ottawa, Ottawa, ON Canada; ^9^ The Edmond J. Safra Program in Parkinson’s Disease and Morton and Gloria Shulman Movement Disorders Clinic, Toronto, ON Canada; ^10^ Sunnybrook Health Sciences Centre, Toronto, ON Canada; ^11^ Western University, London, ON Canada; ^12^ Northwestern University, Evanston, IL USA; ^13^ Li Ka Shing Knowledge Institute, Toronto, ON Canada; ^14^ Temerty Faculty of Medicine, University of Toronto, Toronto, ON Canada; ^15^ Krembil Brain Institute, University Health Network (UHN), Toronto, ON Canada; ^16^ Tanz Centre for Research in Neurodegenerative Diseases, University of Toronto, Toronto, ON Canada; ^17^ Baycrest Health Sciences Centre, Toronto, ON Canada; ^18^ Keenan Research Centre for Biomedical Science, St. Michael’s Hospital, Toronto, ON Canada

## Abstract

**Background:**

Neuropsychiatric symptoms (NPS) constitute a major challenge for patients with Alzheimer’s disease (AD). We have recently demonstrated that in AD, overall NPS burden is significantly associated with patient function. However, few studies have examined the relationship between specific symptom clusters with neurological biomarkers. Therefore, we identified NPS clusters in AD and explored their association with structural neuroimaging markers.

**Method:**

Participants with AD (N = 111) were included from the Ontario Neurodegenerative Disease Research Initiative (ONDRI). NPS were assessed using the neuropsychiatric inventory questionnaire (NPI‐Q), and symptom clusters were identified through exploratory factor analysis. The Semi‐Automatic Brain Region Extraction (SABRE) pipeline was used to compute regional cortical thickness and subcortical volumes using participant MRI data. We then evaluated correlations between symptom clusters with subcortical volumes and regional cortical thickness.

**Result:**

Factor analysis identified four symptom clusters explaining 62% of the variance. These were labeled as “behavioral” (disinhibition, irritability, motor disturbance, and agitation), “psychotic” (hallucinations, delusions, and euphoria), “neurovegetative” (apathy and appetite), and “affective” (depression, anxiety, nighttime behavior) clusters.

The psychotic cluster was associated with increased cortical thickness in bilateral frontal regions, the left inferior parietal lobe (r=0.23, p=0.02), and with left anterior cingulate volumes (r=0.21, p=0.03). The neurovegetative cluster was associated with reductions in volume among bilateral frontal and right‐sided temporal and parietal regions. The affective cluster was associated with increased left anterior cingulate volume (r=0.21, p=0.03).

**Conclusion:**

NPS symptom clusters in AD separate into behavioral, psychotic, neurovegetative, and affective dimensions. These symptom groups demonstrate unique associations with neuroimaging markers.

